# Using feedback in pooled experiments augmented with imputation for high genotyping accuracy at reduced cost

**DOI:** 10.1093/g3journal/jkaf010

**Published:** 2025-01-23

**Authors:** Camille Clouard, Carl Nettelblad

**Affiliations:** Division of Scientific Computing, Department of Information Technology, Uppsala University, Uppsala SE-751 05, Sweden; Division of Scientific Computing, Department of Information Technology, Uppsala University, Uppsala SE-751 05, Sweden; SciLifeLab, Science for Life Laboratory, Uppsala University, Uppsala SE-751 05, Sweden

**Keywords:** SNP array, pooling, imputation, iterative refinement

## Abstract

Conducting genomic selection (GS) in plant breeding programs can substantially speed up the development of new varieties. GS provides more reliable insights when it is based on dense marker data, in which the rare variants can be particularly informative. Despite the availability of new technologies, the cost of large-scale genotyping remains a major limitation to the implementation of GS. We suggest to combine pooled genotyping with population-based imputation as a cost-effective computational strategy for genotyping SNPs. Pooling saves genotyping tests and has proven to accurately capture the rare variants that are usually missed by imputation. In this study, we investigate adding iterative coupling to a joint model of pooling and imputation that we have previously proposed. In each iteration, the imputed genotype probabilities serve as feedback input for adjusting the per-sample prior genotype probabilities, before running a new imputation based on these adjusted data. This flexible setup indirectly imposes consistency between the imputed genotypes and the pooled observations. We demonstrate that repeated cycles of feedback can take advantage of the strengths in both pooling and imputation when an appropriate set of reference haplotypes is available for imputation. The iterations improve greatly upon the initial genotype predictions, achieving very high genotype accuracy for both low- and high-frequency variants. We enhance the average concordance from 94.5% to 98.4% at limited computational cost and without requiring any additional genotype testing.

## Teaser

For large-scale settings, such as plant breeding, acquisition of dense genotypes can constitute a cost challenge. Sometimes, less dense SNP microarrays are used to reduce cost, augmented by imputation to a denser SNP panel. Clouard and Nettelblad instead propose to pool samples and test directly on fewer instances of a more dense array. The authors use a feedback approach to impute individuals, but then make sure that the imputation results are fully consistent with the observations on the pool level. If this does not happen, the prior probabilities are adjusted. They manage to increase accuracy from 94% to 98%, making pooling competitive.

## Introduction

Modern plant breeding programs have increasingly involved genetic data, typically the genotypes of single-nucleotide polymorphisms (SNPs), for supporting the selection process and accelerating the development of new varieties. Related methods such as genomic selection (GS) and genome-wide association studies (GWAS) are more accurate if using high-density marker data The quality of data collected from, e.g. SNP microarrays directly impact the accuracy of the genomic predictions in GS (Gorjanc *et al.* 2017; de Oliveira *et al.* 2020; Gonen *et al.* 2021). While they are restricted to already known markers and not suitable for variant discovery studies, SNP microarrays have remained popular for routine genotyping, and they can provide more accurate calls (He *et al.* 2011) than sequencing techniques. GS and GWAS gain in statistical power with larger population sizes, but genotyping more samples at high density also leads to more expensive data collection. Despite their cost-effectiveness, imputation methods for augmenting low-density genotype data to high density have a known weakness at capturing rare variants, that is with a minor allele frequency (MAF) <5%, while these variants can have a great significance in GS.

We have previously explored the combination of pooling and imputation by simulating SNP chip pooling experiments based on available genotype data of recombinant inbred lines of bread wheat (*Triticum aestivum*). We found that decoding pooled experiments with a nonadaptive and overlapping design, followed by data augmentation via imputation with a suitable method, can improve the genotyping results for rare variants compared with conventional imputation workflows using less dense arrays. Our strategy can cut the number of required SNP array plates by half, compared with nonpooled testing of each sample.

Earlier studies (He *et al.* 2011; Hormozdiari *et al.* 2012) with human data have proposed similar hybrid approaches for genotyping and investigated the sequencing of overlapping DNA pools in conjunction with imputing genotypes assayed on microarrays, and their findings are similar to ours. In their scenarios, sequencing pools instead of individual samples reduces the experimental cost for genotyping, for instance the cost for library preparation can be divided by 3 (Hormozdiari *et al.* 2012). Combining the genotypes decoded from the pooled sequenced reads to the imputed data results in highly accurate genotyping for SNPs with both low and high allele frequency. The pooling scheme takes advantage of the sparsity of variants with low frequency usually missed by imputation methods. Pooling generates very noisy outcomes for common variants due the information theoretic bounds (He *et al.* 2011), but these variants can be accurately imputed thanks to models that take into account the correlation structure between different genetic variants at the population level. The key to these combined genotyping approaches was the use of a likelihood framework that has a greater flexibility than a purely combinatorial approach. Such frameworks allows for carrying the information from imputation, e.g. the linkage disequilibrium (LD) between consecutive markers as well as the MAF, over to the procedure for decoding the pools. He *et al.* (2011) proposed to compute the genotype likelihoods from three different perspectives and take their product as a composite likelihood for calculating the final estimate of each most likely genotype at each entry. Hormozdiari *et al.* (2012) studied an iterative refinement of the imputed genotypes with approximate gradient descent based on a maximum a posteriori (MAP) strategy which handles the minor and the major alleles separately. Both studies demonstrate the performance of injecting information from imputation into the decoding procedure and these results have motivated the approach we present here.

This paper explores what gains in genotyping accuracy can be achieved if we enforce consistency between the pooled observations and imputed genotypes in a scheme similar to the one we have proposed previously (Clouard and Nettelblad 2024). We suggest an iterative coupling procedure between local pattern-consistent decoding and population-based imputation in a likelihood framework. At every iteration, we use the imputed data as feedback for applying a correction to the decoded genotype likelihoods before passing these corrected prior likelihoods to another round of imputation.

We consider these corrections to be similar to any closed-loop control problem, i.e. we seek the weights that optimize our desired goal, which is that the posterior genotypes produced by imputation when reconstituted into pools will match the observed data. There is no direct probabilistic interpretation of the updated priors, and the final output is derived from the posteriors. Using our coupled model, we increase the genotype concordance by 3.9 percentage points, or conversely reduce the genotype discordance by over 70%. We also explore the computational performance of our simulations and experiments. We do not conduct either any detailed quantification of the costs associated with this structure, but we believe that our strategy can be a valuable addition to existing schemes for cost-effective genotype reconstruction at scale, such as nonpooled low-coverage sequencing followed by imputation.

## Materials and methods

We simulate pooled experiments with genotype data of SNPs in an identical fashion to our previously published protocol (Clouard and Nettelblad 2024). Briefly, 496 samples randomly chosen from the recombinant inbred lines were distributed into blocks of 16 samples each. Within each block, the 16 individuals can be arranged as a square, with 4 columns and 4 rows. Each column and each row represents a pool, for which we can simulate the observed pooled genotype. Thanks to each sample being part of two distinct pools, for many variants the identity of any minor allele carriers can be identified directly, especially for variants with a low minor allele frequency. In other cases, the resolution is ambiguous, explaining the need for the following imputation step. There, we used the 16 founders of the population. While our data were inbred, the imputation model used did not assume this. However, we could empirically conclude that no heterozygotes were ever imputed when supplied with the input data, or in later feedback steps.

We consider the results from our pooling simulation to be experimental observations analyzed in later stages of the workflow. The initial decoding algorithm we use computes estimates of the genotype probabilities at any variant and in each sample via expectation maximization. This step is agnostic for LD and allele frequencies at the population level since each variant and each pooling block are treated independently.

**Fig. 1.**
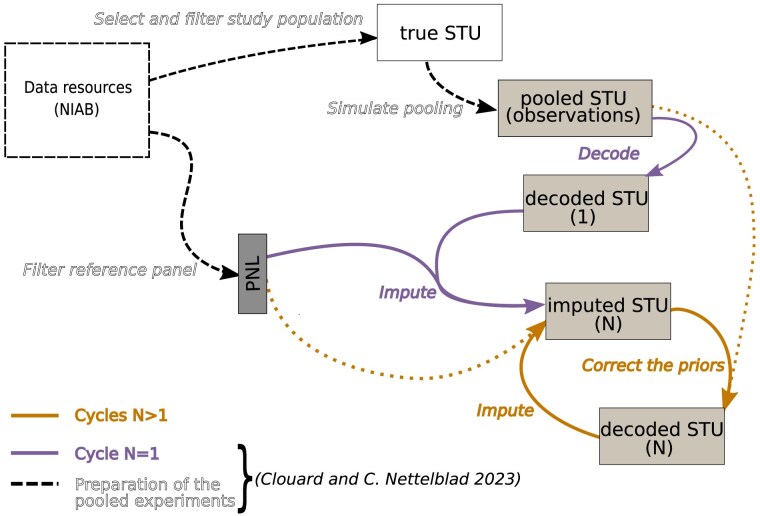
Experimental steps of the iterative genotype pooling and imputation strategy. The data resources used are provided in open access by UCL and were developed by the National Institute for Applied Botany (NIAB) (Scott *et al.* 2021). They consist in a mapping population named *NIAB Diverse MAGIC wheat population* for one thing, and of the set of 16 founders from which the inbred lines were derived. We use the set of founders as reference panel and randomly select 496 samples in the mapping population to serve as study population for our pooling and imputation simulations. The preparation of the pooled experiments and the cycle N=1 are executed with a workflow we developed (Clouard and Nettelblad 2024). The reference panel is assumed to be individually fully genotyped while the study population is mixed into pools prior to genotyping. A block consisting of 16 samples can be arranged into a square, each row and each column forming a pool. Thus, each sample is found in exactly two pools. An example of such a block is shown in Supplementary Fig. S1.1. The pooled genotyping results are decoded with a statistical inference method to individual genotype likelihoods, which are in their turn passed to our imputation algorithm. The later cycles N>1 are the focus of the research in this manuscript. We want to investigate whether the additional iterations of correction of the decoded genotype likelihoods, followed by imputation from the corrected values, can improve the overall accuracy of genotyping in the study population (*imputed STU (N)*) with our pooling strategy. The correction of the priors in the decoded data from the cycles *N* to N+1 is based on a comparison between the likelihood of detecting each allele in the pools after the initial pooling (N=1), and likewise in the simulated the pools from the imputed data in cycle *N*. Such a mechanism enables for incorporating extrinsic information forwarded through the population-based imputation model into the decoding model which is otherwise local to each variant and each pooling block.

Coalescence-based imputation, as we conducted in a second step, is expected to indirectly enrich the genotype probabilities estimated from the pools with information about the global genetic structure in the population. Imputation reconstructs the sequence of genotypes for each sample as a mosaic of the available template haplotypes, which enforces similarity of the genetic profiles across the study population and the reference panel. There is no guarantee that the imputed genotypes are consistent with the decoded prior genotypes passed as input, however. We therefore propose to iteratively apply point-wise corrections to the decoded genotype prior probabilities based on the imputed posterior results, in order to ensure that the imputed per-sample genotypes, which take the global reference into account, are also consistent with the actual pool observations. Such sequential feedback schemes between two models have been implemented in various other research fields. A diagram of our iterative procedure is presented in Fig. 1.

### Experimental data

The cohort of 496 samples used as study population in imputation is part of the *NIAB Diverse MAGIC wheat* population (Scott *et al.* 2021) of inbred lines, and the 16 founders of this experimental population form the reference panel. The genotype data we are looking at, both in the study population and in the reference panel, were originally obtained with sequencing technologies. The authors of the dataset applied strong filtering of the variants that were sequenced, active removal of heterozygous data, and imputation of the missing genotypes in the inbred lines (Scott *et al.* 2021), such that we have considered that the remaining tag SNPs form a set of markers segregating per subgenome that could be used, after proper testing, for developing a microarray. Therefore, we have treated the genotype data as if it would derive from a hypothetical SNP chip of 55K markers, and we have focused on the loci on chromosome 1.

While bread wheat is formally allohexaploid, it can typically be modeled as a diploid-like species (Ma and Gustafson 2005) due to its subgenome structure that consists in hybridized diploid subgenomes, ignoring rare recombinations between them. After additional filtering of the set of markers present on chromosome 1A, the study and reference data sets comprise 1,170 bi-allelic positions.

### Initial round of pooling and imputation

The initial cycle (N=1) consists of a first step of pattern-consistent decoding of the observations at every marker into individual genotype likelihoods, which are denoted with the vector $y^{\left(1\right)}$. In this first decoding step, we assume error-free genotyping, that is, alleles in the pools are detected with full certainty. In a second step, the genotype likelihoods in $y^{\left(1\right)}$ are normalized and used as prior genotype probabilities by a population-based imputation method called *prophaser* (Ausmees and Nettelblad 2022), which predicts the vector of posterior genotype probabilities $z^{\left(1\right)}$ for each variant. For details about the algorithm implemented for pattern-consistency decoding, the imputation methodology, and for a detailed analysis of the imputation performance on pooled data, we refer to our earlier work (Clouard and Nettelblad 2024). We found that common variants were imputed with far lower accuracy than rare ones, when measured in terms of concordance and cross-entropy, and we also observed a larger inter-marker variance for these metrics at higher MAF.

### Subsequent cycles of decoding and imputation

The strategy for carrying out the subsequent cycles N>1 is to verify whether the imputed data in cycle *N* are consistent with the original simulated pooled observations. This realizes a coupling mechanism between the model used for pool-local decoding, and the population-based imputation model. If an inconsistency is found, the decoded genotype likelihoods of each sample in the pool used in cycle *N* are corrected, and constitute the values used as prior genotype probabilities to imputation in cycle N+1. Consistency is not evaluated on a per-individual basis nor on genotype matches, but from the alleles that are expected to be detected in the pools simulated from the imputed data. That is, imputed data in a pool is consistent with the observations if the same alleles are detected in both cases. Whenever an allele is not detected in the imputed data, whereas it should be observed, a correction is applied to the decoded genotype probabilities. Prior genotype probabilities of samples showing imputed data consistent with the pool observation in a certain variant are not updated. However, because of the overlapping design used, the decoded data in a consistent pool might be changed between consecutive cycles if some samples are present in other pools that are inconsistent. An example of such a case is presented in Supplementary File S1 through Supplementary Figs. S1.1 and S1.2, as well as Supplementary Tables S1.1–S1.3.

#### Reconstitution of individual genotype posteriors into pool posteriors

We use a modified version of our original decoding algorithm used in cycle N=1, that we call *repool*. The modified algorithm compares in each cycle the likelihoods of detecting the alleles for a given variant in the imputed data against what alleles should be detected according to the simulated pools. For instance, an observed pooled genotype value equal to 0 means that the probability of detecting the allele $A_{0}=0$ in the pool is 1.0, and the probability of detecting the allele $A_{1}=1$ is 0.0. If the initial genotype of a pool is assayed as equal to 1, both the probability of detecting the allele $A_{0}$ and the probability of detecting allele $A_{1}$ are equal to 1.0.

We denote any sequence of enumerated genotype data *x*, the decoded data in cycle *N*$y^{\left(N\right)}$, and the imputed data in cycle *N*$z^{\left(N\right)}$. *x* is represented as a vector of integer genotype values in {0,1,2}, $y^{\left(N\right)}$ as a vector of log-genotype likelihoods, and $z^{\left(N\right)}$ as a vector of predicted genotype probabilities. The procedure applied for point-wise correction of the decoded outcomes in cycle N>1 in a single pooling block for a single variant is described in Algorithm 1. For a detailed example of calculations with this algorithm, we refer the reader to Supplementary File S1.

In the first part of the algorithm on line 2–12, the probability of detecting the allele $A\in \{A_{0},A_{1}\}$ in the pool *p* from the imputed data $z_{\;p}^{\left(N\right)}$ is computed in two steps. This calculation is done assuming the individual posteriors to be independent. This is done since genotype probabilities per variant per individual are typically treated as independent in downstream analysis tasks, whether they are used to call a single genotype, compute dosage, or in some other way. The goal of our feedback step is to reconcile the implications from such an independent distribution of individual genotypes with the observed combined genotype of the pools.

First, the probability of any enumerated sequence of genotypes $x_{\;p}^{\left(k\right)}$ is the product of the individual imputed genotype probabilities. Second, the likelihood of detecting any allele in each enumerated sequence is determined as the allele contribution from the composite genotype of $x_{\;p}^{\left(k\right)}$, weighted by the likelihood of the sequence. Among the enumerated sequences, some might be inconsistent with the observations, as the two examples provided in Supplementary File S1. It is important to note here that computing the likelihood of detecting an allele is not the same as computing the expected allelic dosage from imputed data. In our algorithm, both homozygotes for the allele *A* and heterozygotes are assumed to equally contribute to detecting the allele *A* in a pool.

The second part of the algorithm on lines 13–22 performs an evaluation of the consistency for each allele separately, and, if necessary, performs a correction for the decoded genotype likelihoods that carry the allele. Likelihoods are only changed to the extent that the imputed likelihoods $Pr(x_{\;p}\;|\;z_{\;p}^{\left(N\right)})$ imply inconsistency with the observation in terms of what alleles would be present. If the data are consistent, no change is made between cycle *N* and N+1 (line 15).

It should be noted that consistency is distinct from correctly imputed results. An inconsistent imputation is definitely incorrect for at least one individual in the pool, but a consistent imputation might still be incorrect for one or more individuals. If the imputed data in the pool *p* are evaluated as inconsistent with the observation, e.g. the allele A is not detected in the imputed samples but should be, the deviation is computed as the entropy of detecting the allele A in the imputed data against detecting it in the observation (line 18). In other words, the deviation is proportional to $Pr(A\;|\;z_{\;p}^{\left(N\right)})$. The deviation is smaller for a higher value for the probability of detection $Pr(A\;|\;z_{\;p}^{\left(N\right)})$. The correction applied to the genotype likelihoods compensates for this deviation, weighted by a dampening factor *w* (line 18). This factor lets us adjust the strength of the correction between individual cycles. Too high values can easily result in overcorrection, since the prior for multiple linked markers in the same individual could be updated in a single cycle, causing an overshoot. A higher dampening factor will make the iterative coupling converge in fewer cycles, but with a lower final accuracy in the predicted genotypes.

**Algorithm 1:** Pseudocode for point-wise reinforcement/correction of the genotype prior probabilities from cycle *N* to cycle N+1 in 1 pooling block for 1 variant

**Require:** *x* is a sequence of integer genotypes, and $y^{\left(N\right)}$ resp. $z^{\left(N\right)}$ are the decoded resp. imputed genotype data in cycle *N*
1: **for** each row pool, column pool *p*^1^
2: L(A)=0, and, respectively $L(\bar{A})=0$ denotes the likelihood of detecting (resp. not detecting) allele $A\in \{A_{0},A_{1}\}$ in the pool *p* based on the imputed genotypes
3: **for all***k* ordered sequences of genotypes $x_{\;p}^{\left(k\right)}$**do**^2^
4: $Pr(x_{\;p}^{\left(k\right)}|z_{\;p}^{\left(N\right)})=\prod_{i=1}^{4}Pr(x_{i}^{\left(k\right)}|z_{i}^{\left(N\right)})$ for each sample *i* part of the pool *p* of size 4
5: **if** pool *p* has genotype $\left(A_{0},A_{0}\right)$ given $x_{\;p}^{\left(k\right)}$**then**
6: $L(A_{0}{)}^{\left(k\right)}=Pr(x_{\;p}^{\left(k\right)}|z_{\;p}^{\left(N\right)})$, and $L(\bar{A_{1}}{)}^{\left(k\right)}=Pr(x_{\;p}^{\left(k\right)}|z_{\;p}^{\left(N\right)})$
7: **else if** pool *p* has genotype $\left(A_{1},A_{1}\right)$ given $x_{\;p}^{\left(k\right)}$**then**
8: $L(A_{1}{)}^{\left(k\right)}=Pr(x_{\;p}^{\left(k\right)}|z_{\;p}^{\left(N\right)})$, and $L(\bar{A_{0}}{)}^{\left(k\right)}=Pr(x_{\;p}^{\left(k\right)}|z_{\;p}^{\left(N\right)})$
9: **else**
10: $L(A_{1}{)}^{\left(k\right)}=Pr(x_{\;p}^{\left(k\right)}|z_{\;p}^{\left(N\right)})$, and $L(A_{0}{)}^{\left(k\right)}=Pr(x_{\;p}^{\left(k\right)}|z_{\;p}^{\left(N\right)})$
11: **for** allele *A* in $\left\{A_{0},A_{1}\right\}$**do**
12: $Pr(A|z_{\;p}^{\left(N\right)})=\frac{\sum_{k}L{\left(A\right)}^{\left(k\right)}}{\sum_{k}L{\left(A\right)}^{\left(k\right)}+L(\bar{A}{)}^{\left(k\right)}}$
13: **if**$Pr(A|z_{\;p}^{\left(N\right)})<1.0$**and**Pr(A∈p)>0.0**then**^3^
14: **for** each sample *i* in the pool *p***do**
15: **for** genotype *g***in**$\left\{(A_{0},A_{0}),(A_{0},A_{1}),(A_{1},A_{1})\right\}$**do**
16: $y(g{)}_{i}^{\left(N+1\right)}=y(g{)}_{i}^{\left(N\right)}-log(Pr(A|z_{\;p}^{\left(N\right)}))\cdot w$**if**A∈g^4^

*Notes:*

1 Since the pooling design is overlapping and has a degree of intersection equal to 2, each sample is processed twice: once in a row pool, and once in a column pool.

2 There are at most 3^4^ sequences to marginalize over in the theoretical case with 1 heterozygous and 2 homozygous genotypes possible for each of the 4 samples in the pool. In the case of the inbred lines of wheat we handle that are fully homozygous, the largest number of sequences is 2^4^.

3 If the probability of detecting a given allele in the imputed data is less than 1, but this allele is detected in the observations, the imputation outcome is not considered consistent. The magnitude of the deviation in the imputed data are used in the output step to increase the prior for the missing allele in $y{\left(g\right)}^{\left(N+1\right)}$.

4 We denote *w* the dampening factor for the pooled outcomes applied at every cycle, and $-log(Pr(A|z_{\;p}^{\left(N\right)}))$ is the correction term. In practice, we apply a max-transformation such that the correction term has a lower bound set to $-log(10^{-3})$. If $Pr(A|z_{\;p}^{\left(N\right)})$ is close to 1.0, the correction applied is almost 0. In other words, the higher the inconsistency between the imputed and the initially decoded data is, the more the decoded genotype likelihoods are corrected.

#### Imputation with *prophaser*

Once the priors have been correcter, *prophaser* is executed again, with the only difference being the new priors. The same reference panel, the same genetic map, and the same hyperparameters, e.g. an effective population size equal to 16, are used.

#### Technical implementation of the iterations and computational resources

Data downloading and preprocessing, simulation of pooling, as well as all cycles of decoding and imputation, are executed on a cluster node HP ProLiant SL230s Gen8 that has a memory configuration of 128 GiB and consists of 2 eight-core CPUs (Intel Xeon E5-2660).

We extend our workflow (Clouard 2023) by adding a wrapper Bash script which consists of chained Slurm jobs with job dependencies, such that a new cycle of decoding and imputation starts only if the previous one has successfully completed for all samples.

### Metrics for analyzing the genotyping accuracy and the computational performance

As we are interested in comparing the accuracy of the genotype posteriors from later cycles against the results obtained in the cycle 1, we use the same metrics as in our first study (Clouard and Nettelblad 2024), that is the genotype concordance and the cross-entropy averaged per variant in the study population and presented as the average per binned MAF, with bins of size 0.01. We recall that the genotype concordance measures the degree of similarity between the true and the predicted genotype represented as integers, it is equal to 1 if there is no difference between the true and the predicted value. The cross-entropy renders the elementwise distance between the true and predicted genotype probabilitiesIt is equal to 0 if the ground truth and the prediction are identical.

In order to get more detailed insights into what markers are corrected in any cycle, we compute separately the marker-wise genotype concordance and cross-entropy for the entries with modified priors on the one hand, and for the unchanged data on the other. In this case, there is no averaging per variant before averaging per MAF-bin as this would be less relevant given that there is a variable number of entries that have a changed prior at each locus. We consider a prior to be significantly modified between two cycles if the difference of the prior log-likelihood is larger than $\epsilon =10^{-5}$.

Last, we measure the time and memory usage for computation, both for the correction of the priors in the full study population, and for the per-sample imputation. Measuring the global execution time for all cycles is less relevant, since the time used for the orchestration of the full set of chained jobs with dependencies will mostly be dependent on the load level and size of the shared computational cluster used.

## Results

In total, 42 cycles are run. The dampening factor *w* for the genotype likelihood updates is set to 0.01 in the results presented and discussed, but supplementary results exploring the effects of other values for *w* can be found in Supplementary File S2, specifically Supplementary Figs. S2.1–S2.10, and Supplementary Table S2.1. w=0.01 turned out to offer nearly as good results as lower values, but with convergence attained in fewer cycles. We therefore focus on presenting results acheived while using that value here.

### Impact of additional cycles of correction and imputation on the accuracy of genotyping

**Fig. 2.**
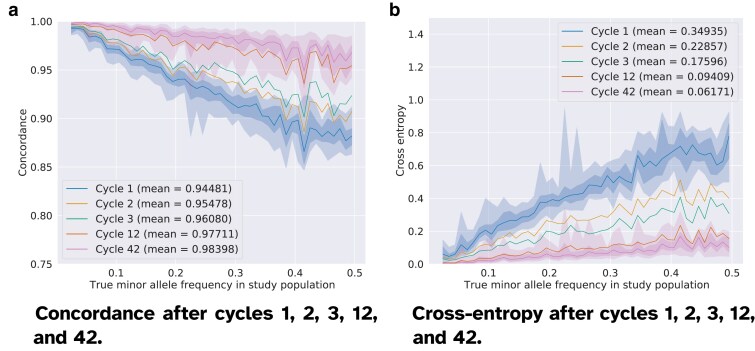
Genotyping accuracy after cycles 1, 2, 3, 12, and 42 (dampening factor w=0.01). The concordance and cross-entropy scores are computed between the imputed data (*imputed STU (N)* in Fig. 1) and the filtered data (*true STU*). The imputed markers are sorted per ascending true MAF in the study population and categorized into MAF bins (bin size equal to 0.01). Each marker has a smoothed concordance and cross-entropy score which is calculated as the average value in a rolling window of 5 MAF-consecutive markers. The solid line shows the median accuracy value (concordance or cross-entropy) in each MAF bin, and the shadowed areas represent the quantiles [0.0,0.01,0.25,0.75,0.99,1.0]. For the sake of readability, the envelopes for the quantiles are shown only for the first and the last cycles. We observe the strongest improvement in genotyping accuracy for variants with MAF≥0.3 and through the first cycles (2 and 3). The increase in accuracy is more limited in later cycles, as the model converges to imputation results consistent with the pool observations. The irregularities of the median line and of the envelopes, for instance around MAF≃0.22, are due to the sparsity of markers in some parts of the MAF spectrum. a) Concordance computed for all genotypes. b) Cross-entropy computed for all genotypes.

In Fig. 2, we show the average concordance and cross-entropy with respect to the MAF after the cycles 1, 2, 3, 12, and 42. We observe an overall improvement in both metrics, which confirms that repeated corrections and imputations increase the genotyping accuracy. The average concordance across all makers rises from 94.5% (N=1) to 98.4% (N=42). We also note that much of the gain in accuracy occurs already in the first few cycles. This improvement almost 1 percentage point from cycle 1 to cycle 2 but by barely 0.7% in the 31 latest cycles (from N=12 to N=42). The extent of the gains is highly dependent on the MAF. As shown in Table 1, most loci in the set of variants that we study are common variants as 1,094 out of 1,170 loci have a MAF>0.1. As shown in Fig. 2 , most of the improvement from repeated cycles arise for higher MAF values. For the rare variants, we previously found that the concordance is approximately 99% already after one cycle of decoding and imputation (Clouard and Nettelblad 2024). This score is also improved with additional cycles, but to a smaller extent than for the common variants. After 42 cycles, the variants with MAF<0.1 are close to perfectly imputed and the concordance for the variants with the highest MAF has improved by nearly 10 percentage points.

**Table 1.** Statistics for markers per MAF bin in the population of inbred lines (true data).

	0.00–0.05	0.05–0.10	0.10–0.20	0.20–0.30	0.30–0.40	0.40–0.50	Total
Counts	76	260	363	221	139	111	1170
Proportions^a^	0.065	0.222	0.310	0.189	0.119	0.095	1.000

a SNP proportions per MAF bin with respect to the total number of SNPs in the genetic map.

**Fig. 3.**
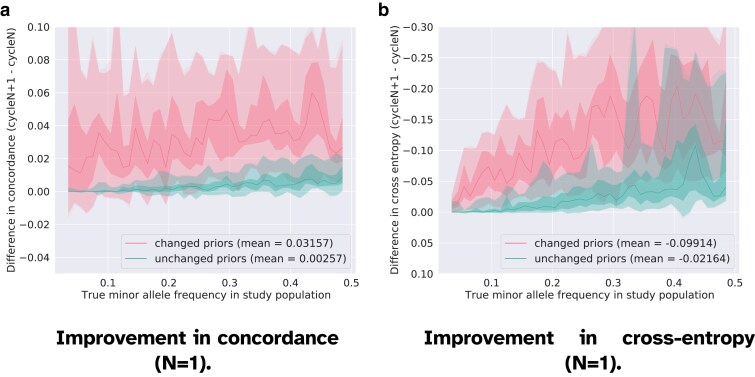
Improvement in genotyping accuracy between cycle 2 and cycle 1 (N=1) computed separately for the genotypes with an *updated* prior and for the genotypes with an *unchanged* prior (dampening factor w=0.01). The solid line shows the median accuracy score in each bin. The shadowed areas display the quantiles [0.0,0.01,0.25,0.75,0.99,1.0]. The largest gain in accuracy is obtained for the set of genotypes whose priors were corrected, but an additional round of imputation also improves, to a smaller extent, the genotyping accuracy for the predicted genotypes that were consistent with the pooled outcomes and that were therefore not changed. In both sets of priors, the greatest improvements are observed for the common variants. The genotyping accuracy of rare variants is slightly improved as well in the case where the priors are updated. a) Positive values indicate that the concordance achieved after the cycle N+1 is higher than the concordance after cycle *N*, that is, the iteration N+1 has improved the genotyping accuracy. b) Negative values indicate that the cross-entropy achieved after the cycle N+1 is lower than the concordance after cycle *N*, that is, the iteration N+1 has improved the genotyping accuracy. The *y*-axis is reverted in order to display the improvements above the *x*-axis.

**Fig. 4.**
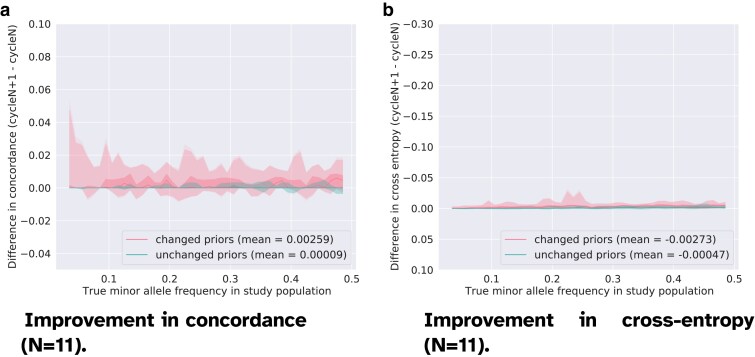
Improvement in genotyping accuracy between cycle 12 and cycle 11 (N=11) computed separately for the genotypes with an *updated* prior and for the genotypes with an *unchanged* prior (dampening factor w=0.01). The solid line shows the median accuracy score in each bin. The shadowed areas display the quantiles [0.0,0.01,0.25,0.75,0.99,1.0]. We still observe small gain in accuracy for the genotypes which have corrected priors, on the contrary to the markers with unchanged priors that seem to have reached optimal values for the predicted genotypes. a) The pink envelope in a positive range suggests that changing some priors still triggers swaps to correct predictions. b) Cross-entropy is barely modified, which indicates that in spite of changes in favor of the correct true genotype, the predictions are weak i.e. the largest genotype probability is close to 0.5.

**Fig. 5.**
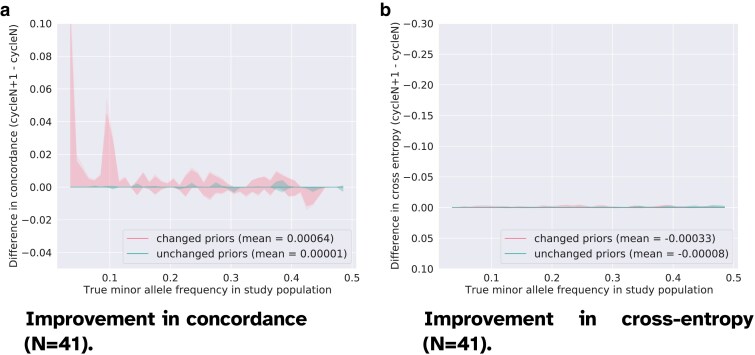
Improvement in genotyping accuracy between cycle 42 and cycle 41 (N=41) computed separately for the genotypes with an *updated* prior and for the genotypes with an *unchanged* prior (dampening factor w=0.01). The solid line shows the median accuracy score in each bin. The shadowed areas display the quantiles [0.0,0.01,0.25,0.75,0.99,1.0]. We no longer observe any significant gain in accuracy that could be correlated to changes in the prior genotype probabilities (on average, concordance is improved by 0.00064). a) We note large positive peaks around MAF=0.1 and MAF=0.02 and a smaller negative peak around MAF=0.42. These peaks suggest a sort of instability in the results, likely due to weak genotype predictions. At some loci and for some individuals, the two homozygotes might have almost identical probabilities, allowing small fluctuations to affect the genotype calls, and, in turn, concordance. b) The cross-entropy score is almost unchanged, which is consistent with convergence being attained, and that the variations in concordance being due to almost identical probabilities for opposite homozygotes in some instances. The tables below show an example of a true genotype value that is changed to the incorrect homozygote in cycle 42 (detrimental swap), and an example of a true genotype value that is changed correctly (favorable swap). We do observe homozygous genotype probabilities that are close to 0.5.

In Figs. 3–5, we present the changes in the genotyping accuracy in early (N=1–2), intermediate (N=11–12), and late (N=41–42) consecutive cycles. We use the same vertical range across the graphs to more clearly show the behavior as we approach convergence. The improvement is computed as the difference between the accuracy in the cycles N+1 and *N*. In these figures, we separate the gain in accuracy between consecutive cycles for the set of genotype entries whose priors were updated by *repool* on the one hand, and those that remained unchanged on the other hand. What set of decoded entries are corrected might vary at each cycle. The priors passed as input to *prophaser* are considered as unchanged if the elementwise difference between the genotype probabilities in cycle *N* and cycle N+1 is less than $\epsilon =10^{-5}$. We observe that the markers with updated priors have the largest increase in genotyping accuracy in all cycles, but that the magnitude of the increases subsides as we approach convergence. The average concordance of markers with changed prior genotype probabilities rises by 0.03157 between the first and the second cycle, and only 0.00064 between the second last and the last cycle. This confirms that the corrections we apply have an actual and positive impact, which is moreover the strongest in the earliest cycles. In Fig. 3, we also note a gain in accuracy for the set of unchanged genotypes that are in the MAF range [0.3,0.5]. This suggests that corrections applied to some genotypes can improve the predictions at unchanged entries, typically through high LD between markers.

**Table:** 

Example of detrimental last swap in genotype prediction with nearly equally likely GP for the opposite homozygotes: Sample A1343_A1343 at variant 1:471263300 (MAF=0.429435) has GT = 0/0, which is correctly predicted after cycle 41 but is predicted to 1/1 in cycle 42.		
	Cycle 41	Cycle 42
GT:GP	0/0:0.513935,0,0.486065	1/1:0.489339,0,0.510661
Concordance	1.0	0.0
Difference cycle 42 − cycle 41	− 1.0	
Cross-entropy	5.59603	5.87920
Difference cycle 42 − cycle 41	+0.28317	

**Table:** 

Example of favorable last swap in genotype prediction with nearly equally likely GP for the opposite homozygotes: Sample A1118_A1118 at variant 1:29607405 (MAF=0.032258) has GT = 1/1, which is incorrectly predicted to 0/0 after cycle 41 but rectified to 1/1 in cycle 42.		
	Cycle 41	Cycle 42
GT:GP	0/0:0.520817,0,0.479183	1/1:0.495494,0,0.504506
Concordance	0.0	1.0
Difference cycle 42 − cycle 41	+1.0	
Cross-entropy	5.99613	5.70459
Difference cycle 42 − cycle 41	− 0.291541	

Table 2 tracks the changes in the imputed probability in the pool with the samples A1060_A1060, A1075_A1075, A1394_A1394, and A1082_A1082, for the variant 1:11814388. After the first cycle of decoding and imputation, all samples, but A1060_A1060, are correctly imputed. A1060_A1060 is first predicted to the opposite homozygote and swaps to the correct prediction after 23 cycles of correction and imputation. In later cycles, this correct call gets stronger as the posterior probability of the homozygous genotype rises from 50.9% after cycle 23 to 69.6% following cycle 42. This change means that the cross-entropy contribution from this variant in this sample continues to drop. The example of A1060_A1060 demonstrates that our method can improve the posterior genotype probabilities gradually. A closer analysis of the decoded genotype likelihoods in Supplementary Table S1.1. reveals that the successive corrections increase the prior likelihood for the allele 1 in the sample A1060_A1060. For sample A1082_A1082, we observe that the posterior genotype probabilities oscillate before converging to the correct prediction. In order to understand this behavior, we need to consider the data in the overlapping pool across cycles. The overlapping pool increases the likelihood for allele 0, due to an inconsistency due to its absence in that pool, resulting in a competing effect on A1082_A1082 from the two pools. The weakening in the correct genotype prediction for samples A1075_A1075 and A1394_A1394 is explained by the successive adjustments that raise the likelihood of genotypes carrying the alternate allele. Nonetheless, the imputed genotypes remain correct.

**Table 2.** Examples of genotypes predicted for four samples in a pool at marker 1:11814388 through several cycles of pooling and imputation (w=0.01).

	A1060_A1060			A1075_A1075			A1394_A1394			A1082_A1082		
True genotypes	0.0	0.0	1.0	1.0	0.0	0.0	1.0	0.0	0.0	1.0	0.0	0.0
Cycle 1												
Imputed genotypes	0.99989	0.0	0.00011	1.0	0.0	0.0	0.999892	0.0	0.000108	0.489336	0.0	0.510664
Cycle 2												
Imputed genotypes	0.999824	0.0	0.000176	1.0	0.0	0.0	0.999828	0.0	0.000172	0.657397	0.0	0.342603
Cycle 11												
Imputed genotypes	0.938863	0.0	0.061137	0.999998	0.0	0.000002	0.954346	0.0	0.045654	0.50544	0.0	0.49456
Cycle 12												
Imputed genotypes	0.907764	0.0	0.092236	0.999998	0.0	0.000002	0.933557	0.0	0.066443	0.493936	0.0	0.506064
Cycle 22												
Imputed genotypes	0.514556	0.0	0.485444	0.999971	0.0	0.000029	0.734207	0.0	0.265793	0.575863	0.0	0.424137
Cycle 23												
Imputed genotypes	0.491217	0.0	0.508783	0.999966	0.0	0.000034	0.728201	0.0	0.271799	0.587209	0.0	0.412791
Cycle 41												
Imputed genotypes	0.307134	0.0	0.692866	0.999513	0.0	0.000487	0.705797	0.0	0.294203	0.69339	0.0	0.30661
Cycle 42												
Imputed genotypes	0.304208	0.0	0.695792	0.999441	0.0	0.000559	0.704606	0.0	0.295394	0.696172	0.0	0.303828

True genotypes and imputed genotypes are given as genotypes probabilities. For sample A1060_A1060, the correct genotype was called once cycle 23 had completed. Sample A1060_A1060 in this example has the true genotype 1/1 (homozygote for the alternate allele, that is (0.0,0.0,1.0) if written as genotype probabilities. The first round of imputation predicts with high probability the genotype of all samples but A1060_A1060 correctly, which has a probability 0.999 to be homozygote for the reference allele. If applying pooling and decoding from these genotype predictions in cycle 1 to sample A1060_A1060 , the log10 likelihoods of the genotypes carrying the allele 1 in the pooled data are hence updated to −12+0.0067=−11.9933 and −0.30103+0.006718=−0.294312. While A1060_A1060 benefits from the iterative corrections and is eventually imputed to the correct genotype with probability 0.696, the results for sample A1394_A1394 are worsened. The final genotype prediction after cycle 42 has completed is still correct, however the associated probability is lower (0.704606 against 0.999892 after cycle 1). This trend is likely due to the successive corrections of the decoded genotypes that increase the likelihood of the alternate allele for all samples in the pools. The first prediction for sample A1082_A1082 is weakly wrong since the opposite homozygoteis estimated as almost equally likely. The genotype imputed seems to oscillate in the early cycles, but is eventually correct. This is due to contributions from the other pool where A1082_A1082 is included, where an inconsistency in a lack of the reference allele causes prior updates to increase the probability for that allele (correctly), while the inconsistency in the pool illustrated here, due to the lack of an alternate allele, is driving the prior into the wrong direction, until the posterior in A1060_A1060 has risen high enough to reduce those contributions. This example of samples and locus illustrates how our strategy can improve genotype concordance for samples by enforcing consistency with the pooled observations, but also showcases some of the challenges involved in applying gradual modifications to find the most parsimonious resolution.

In Fig. 5, there are some distinct outliers in concordance in the last cycle, whereas the cross-entropy is uniform and almost equal to 0. This difference can be due to the fact that the concordance is based on hard genotype calls, where a probability transition across 0.5 can have an outsized effect. When the uncertainty is large, several individuals in a pool can stay at approximately 0.5 even at convergence. For instance, a change in the genotype probability from Pr(G=2)=0.479 to Pr(G=2)=0.505 in the example of favorable swap results in an obvious peak down, but has negligible impact on the cross-entropy.

Table 3 gives counts of genotypes corrected between consecutive cycles. The counts across all samples are aggregated within MAF intervals, such that we find that for the rare variants, about 2,100 genotypes are updated in each cycle, that is 5.6% of the 37,696 data points in the interval. This low percentage suggests that the posterior genotypes after imputation are mostly consistent with the pooled observations. The MAF intervals that span over 0.2 to 0.5 have the largest shares of corrected genotypes with a range from ca. 32% up to nearly 40%. The counts of corrected genotypes slightly vary through the cycles, for instance, in the MAF interval [0.4,0.5], 35.9% of the genotypes are updated between the cycles 1 and 2, and 39.8% between the cycles 41 and 42. In contrast, the MAF interval [0.05,0.1] shows the opposite trend with 13.4% of corrected genotypes from the cycle 1 to 2, and eventually 11.9% from the cycle 41 to 42. Across all variants and samples, about 25% of the data points are updated after each cycle.

**Table 3.** Statistics for genotypes (variants × samples) per MAF bin in the population of inbred lines (pooled data, dampening factor for the pooled genotype likelihoods equal to 0.01).

	0.00–0.05	0.05–0.10	0.10–0.20	0.20–0.30	0.30–0.40	0.40–0.50	Total
*All cycles: pooled data*							
Counts^a^	37696	128960	180048	109616	68944	55056	580320
Proportions^b^	0.065	0.222	0.310	0.189	0.119	0.095	1.000
*Cycle 2 vs. cycle 1: genotypes with updated priors in pooled data*							
Counts	2108	17220	44845	35577	24517	19782	144049
Proportions w.r.t. the bin	0.056	0.134	0.249	0.325	0.356	0.359	0.248
*Cycle 3 vs. cycle 2: genotypes with updated priors in pooled data*							
Counts	2073	16935	44873	35452	24686	20406	144425
Proportions w.r.t. the bin	0.055	0.131	0.249	0.323	0.358	0.371	0.249
*Cycle 12 vs. cycle 11: genotypes with updated priors in pooled data*							
Counts	2133	16558	45382	36049	25885	21820	147827
Proportions w.r.t. the bin	0.057	0.128	0.252	0.329	0.375	0.396	0.255
*Cycle 42 vs. cycle 41: genotypes with updated priors in pooled data*							
Counts	2073	15304	43018	34984	25647	21931	142957
Proportions w.r.t. the bin	0.055	0.119	0.239	0.319	0.372	0.398	0.246

a The count of genotypes correspond to the number of variants in the MAF-interval multiplied by the number of samples in the study population. For instance, Table 1 indicates that 76 variants have a MAF <5%, which correspond to the genotype count equal to 76×496=37,696.

b SNPs proportions per MAF bin with respect to the total number of SNPs on the genetic map.

The overall stability in the number of corrected genotypes after each marker observed in Table 3 might look discordant with the results in Fig. 2, where the gain in accuracy after correction and imputation decreases through the cycles. Therefore, we calculate the total correction that is added to the pooled decoded data in each cycle, and we observe that the total correction in the decoded genotypes drops through the cycles (Supplementary Fig. S2.10). That is, although the number of genotypes that are corrected is approximately the same in any cycle, the amplitude of the correction per genotype decreases strongly over the iterations.

These gradually smaller corrections, as well as the trend in Figs. 2–5, indicate a convergence of our coupled model.

Any imputation scheme is sensitive to the completeness of the reference set used. In this study, we employed the fact that the 16 founders by definition represent all haplotypes found within the recombinant inbred lines. To understand the behavior when the reference set is incomplete, we ran an auxiliary experiment with only eight reference samples, leaving out eight of the founders completely. Since the intent of the NIAB Diverse MAGIC wheat population was to have diverse founders, this reduced imputation performance. In cycle 1, the concordance was only 89.6%, relative to 94.5% with 16 founders. With the lack of proper haplotypes, the feedback mechanism also suffered, with slower convergence. In cycle 2, the concordance only improved marginally to 89.7%, to reach 90.8% in cycle 12, and 91,7% in cycle 42. This shows that the feedback mechanism was still successful in enforcing consistency with the observed genotypes, albeit with slower convergence, while the imputation process as a whole was hampered by the incomplete reference set. Even with this inappropriate reference set, rare variants were imputed well, with the largest errors, and the largest benefits due to feedback, found in the variants within the highest MAF bins.

### Computational performance of the coupled iterations

In any cycle N>1, correcting the decoded data with *repool* takes about 7 s, with <1 GB of memory used. The computational performance of imputation with *prophaser* is similar to the cycle 1, on the order of 1 s of computation time including data loading and saving, and <1 GB of memory use. Hence, from the perspective of the computational complexity, the gains in genotyping accuracy achieved thanks to further cycles of pool reconstitution, prior updates, and imputation have a very low cost. In more typical imputation scenarios with larger reference sets, the time and memory needed for the pool reconstitution step would be unaffected, while the imputation time usage in each cycle would scale in the same way as in a conventional imputation workflow.

## Discussion

We obtain accuracy levels that are in the same as in earlier similar work (He *et al.* 2011; Hormozdiari *et al.* 2012) without parameterizing the distribution of the genotypes based on the Hardy–Weinberg equilibrium (HWE) or using pedigree information. Both *repool* and *prophaser* could handle various levels of heterozygosity in the population. They implicitly model a general diploid population, with a high frequency of heterozygotes, but based on the properties of the observed data, they respect the inbred nature of the processed input population in our dataset. Such flexibility mainly lies in the likelihood framework in which the genotype data are expressed.

The gain in genotyping accuracy achieved through additional cycles indicates that the feedback structure successfully combines the strengths of imputation and pooling, which eventually yields very precise predictions for the genotypes at any variant. The improvements at each cycle confirm that the decoding and the imputation models exploit complementary sources of information, that are the combinatorial constraints local to the pools on the one hand, and the inter- and intraindividual genetic structure on the other. As in other iterative methods, we believe that exploring adaptive values of the dampening factor *w* could contribute to optimize the tradeoff between the gain in accuracy and the number of cycles to execute. Momentum approaches, as frequently used in deep learning, would be one way to achieve this. Using a method such as Adam (Kingma and Ba 2017), treating the current update value (without *w*) as the gradient, would be one promising way to accomplish this. Without external ground truth, the cross-entropy of the posterior genotypes for two successive iterations could be used as a convergence threshold, rather than the fixed cycle count employed in this explorative study.

Regardless of the number of cycles executed, our strategy has the advantage of not requiring any extra sequencing nor testing on microarrays. Instead, we exclusively rely on array data and on the global genetic information carried in the library of reference haplotypes. The computational cost of each additional iteration is mainly driven by the size of the reference panel in the imputation step. That is, with a small reference panel such as the one we have, the time complexity remains very low and executing the imputation on CPU resources only demonstrates sufficient performance. In the case larger reference panels should be investigated, we have also implemented a version of *prophaser* that is suitable for execution on GPU.

Our results with a reduced reference set also reinforces the drastic deterioration of imputation performance that is possible if the haplotypes found in the study individuals are highly divergent from the variability covered by the reference set. While our feedback mechanism as such is successful in handling such scenarios and still providing some improvement to the results, the uncertainty introduced through pooling makes the issue of a proper reference set more crucial than ever.

## Supplementary Material

jkaf010_Supplementary_Data

## Data Availability

The datasets supporting the conclusions of this article are available at http://mtweb.cs.ucl.ac.uk/mus/www/MAGICdiverse/MAGIC_diverse_FILES/ (genotype data) and https://urgi.versailles.inra.fr/download/iwgsc/IWGSC_RefSeq_Annotations/v1.0/iwgsc_refseqv1.0_recombination_rate_analysis.zip (genetic maps). We refer to a workflow which can be found at https://github.com/camcl/poolimputeSNPs/tree/iterpoolimp for reproducing our results and simulations. Note, however, that the cycles of correcting decoding and imputation were run with the computational resources provided by UPPMAX which uses Slurm and specific settings. This implies some (extensive) refactoring of our shell scripts. This workflow uses source codes that are accessible at https://github.com/camcl/genotypooler/tree/iterpoolimp (*genotypooler*) and at https://github.com/scicompuu/prophaser/tree/multilevel (*prophaser*). Supplemental material available at G3 online.
