# Supplementary material for: Using feedback in pooled experiments augmented with imputation for high genotyping accuracy at reduced cost
Source: G3 (Bethesda). 2025 Jan 23;15(3):jkaf010. doi: 10.1093/g3journal/jkaf010 (PMC11917477; doi:10.1093/g3journal/jkaf010)
Supplement: jkaf010_Supplementary_Data [file jkaf010_supplementary_data.zip › File_S1_G3-2024-405589.pdf]

Combining pooling and imputation in a feedback structure for SNP genotyping at reduced cost and increased accuracy

Camille Clouard [1], Carl Nettelblad [1,2*]

[1] Division of Scientific Computing, Department of Information Technology, Uppsala University, Lägerhyddsvägen 1, 75237, Uppsala, Sweden
[2] SciLifeLab, Science for Life Laboratory, Husargatan 3, 75237, Uppsala, Sweden
*Corresponding author: carl.nettelblad@it.uu.se

Supplemental file 1:

- Number of pages: 7
- Number of figures: 2
- Number of tables: 3

`repool`: A decoding algorithm for iterative adjustment of prior probabilities from pool experiments based on feedback from imputation

Introduction

This document provides numerical examples and explanations for the algorithm `repool` presented in the main manuscript.

General idea of the algorithm `repool`

Given the outcomes of pooled genotyping observations on one hand, and the corresponding imputed data on the other hand, `repool` implements a strategy that iteratively adjusts the decoded genotype probabilities by using the imputed genotypes as feedback. The algorithm compares the probability of detecting the alleles in the "repooled" imputed genotypes, based on the imputed posterior probabilities of the genotypes in each individual, with the probability of detecting those alleles in the observed outcomes of pooled genotyping experiments. If the predicted genotypes from imputation are not consistent with the the observations, `repool` modifies the priors in a direction to promote the desired allele. For instance, if allele 0 is unlikely to be detected in the pool, according to the imputed data, whereas the corresponding observation indicates the allele should be detected, `repool` will increase the likelihood of the heterozygous genotype and the homozygous genotype for that allele in the decoded data. In other words, at the next imputation round, the prior probabilities used in the Hidden Markov Model will favor genotypes that contain allele 0, compared to the previous input.

Notations

For any biallelic genetic marker, we denote:

- $\{A_0 = 0, A_1 = 1\}$ are the alleles (e.g. ref/alt), that can be found at this locus,
- x is a sequence of genotypes represented as a vector of integers in $\{0, 1, 2\}$. The genotypes of the samples in the pools P_1 and P_8 form the sequences $x_{P_1} = (x_1, x_2, x_3, x_4)$ and $x_{P_8} = (x_4, x_8, x_{12}, x_{16})$,
- $y^{(N)}$ is the decoded data in cycle N represented as a vector of genotype log-likelihoods,
- $z^{(N)}$ is the imputed data in cycle N represented as a vector of predicted genotype probabilities.

Representation of a pooling block

Figure S1.1 is the representation we use for a pooling block. It explicitly illustrates the overlap between the 4 row pools and the 4 column pools. In this example, we track the

modifications performed on the the genotype probabilities in the first row pool, that is the first 4 samples in our data. We remind that in the case of the NIAB MAGIC wheat population, the samples are inbred and therefore fully homozygous. Our implementation can however handle the case where the samples would be heterozygotes as well.

G_1	G_2	G_3	G_4
G_5	G_6	G_7	G_8
G_9	G_{10}	G_{11}	G_{12}
G_{13}	G_{14}	G_{15}	G_{16}

Figure S1.1: **Representation of a pooling block as a square grid.**
The samples G_1, G_2, G_3, G_4 form pool $\mathbf{P_1}$, which is a row pool. G_4 is also part of the pool $\mathbf{P_8}$ together with G_8, G_{12}, G_{16}, which is a column pool. Several different representations of the genotype G_i of any sample i can be relevant. For instance, if G_4 has the true unphased genotype 1/1, the following are equivalent representations:

- An integer genotype $G_i = \{0, 1, 2\}$: $G_4 = 2$.
- A pair of alleles in $\{A_0, A_1\}$: if $A_1 = 1$, then $G_4 = (A_1, A_1)$.
- A tuple of log10 genotype likelihoods: $G_4 = (-12.0, -12.0, 0.0)$ with infinite values from null likelihoods cut to -12.
- A tuple of genotype probabilities: $G_4 = (0.0, 0.0, 1.0)$.

Example of computations with `repool`

Table S1.1 presents, for the marker at position 11814388 on the chromosome 1, genotype data from the 4th sample in our simulation through the cycles 1, 2, 11, 12, 22, 23, 41, 42. The computations with `repool` were run with the parameter $w = 0.01$. We refer to the main article for the meaning of this parameter. If the alleles detected in the "repooled" imputed data and in the pooled observation are not consistent with each other, some genotype likelihoods in the decoded data are increased, otherwise they are left unchanged. The probability of detecting an allele derives from applying the likelihood theory under the assumption that the genotypes are independent of each other within a sequence. Figures S1.2 and S1.3, as well as Tables S1.2 and S1.3, focus more specifically on the calculations performed with `repool` from cycle 1 to cycle 2 for sample G_4, which is part of the pools P_1 and P_8. These examples render the impact of modifying the genotype value of a sample on the other pools in the block.

Details of the calculations in the first row pool (P_1) in the first pooling block

Table S1.1 shows that the decoded genotype likelihoods are updated between the cycles 1 and 2. However, in Figures S1.2 and Table S1.2, the analysis shows that "repooling" P1 from the imputed data does not impact the decoded genotype of sample 4. Hence, we have to look at what is happening in the column pool P_8 which also involves the sample G_4.

Details of the calculations in the fourth column pool (P_8) in the first pooling block

In Figures S1.3 and Table S1.3, we observe that, in the presence of a prediction with low confidence of nearly equiprobable homozygous genotypes in the pool, the genotype likelihoods are substantially changed. These changes impact G_4, and therefore P_1 as well in the next cycle.

Figures

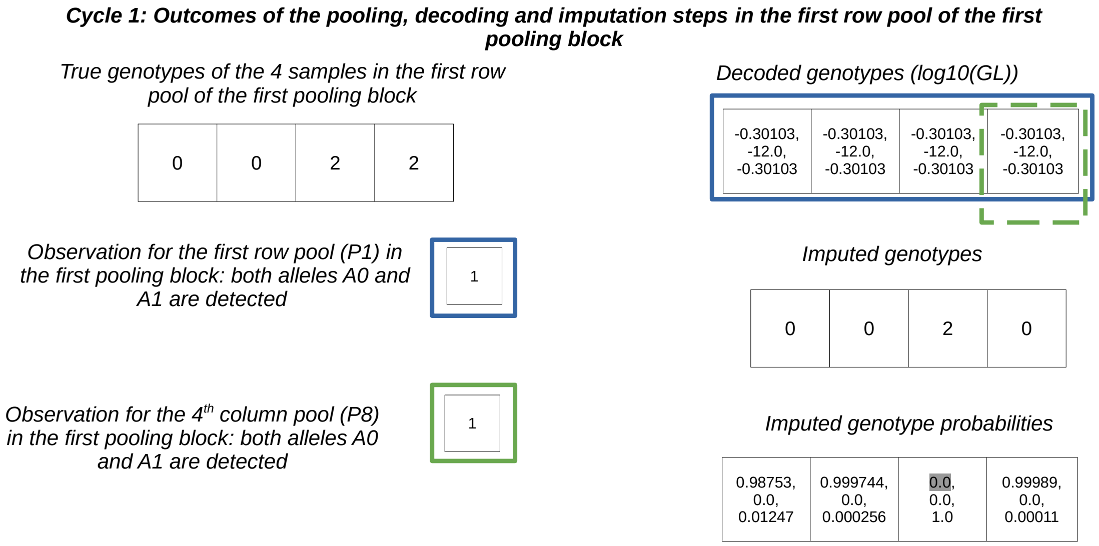

Figure S1.2: **True, pooled, decoded, and imputed genotype data for the pool P_1 in cycle 1.** We implemented a code that simulates error-free pooled genotyping on the true data $(0, 0, 2, 2)$, resulting in genotype 1 for the pool P_1. The imputed data after cycle 1 is $(0, 0, 2, 0)$, which matches the vector of true genotypes for all samples, but G_4. The probabilities of the imputed genotypes are high (> 0.98) and we note that the probability of the heterozygous genotype is zero in all samples. Despite the imputation error, this outcome is consistent with the observed result, since both genotype sequences would result in both alleles being observed in the pool.

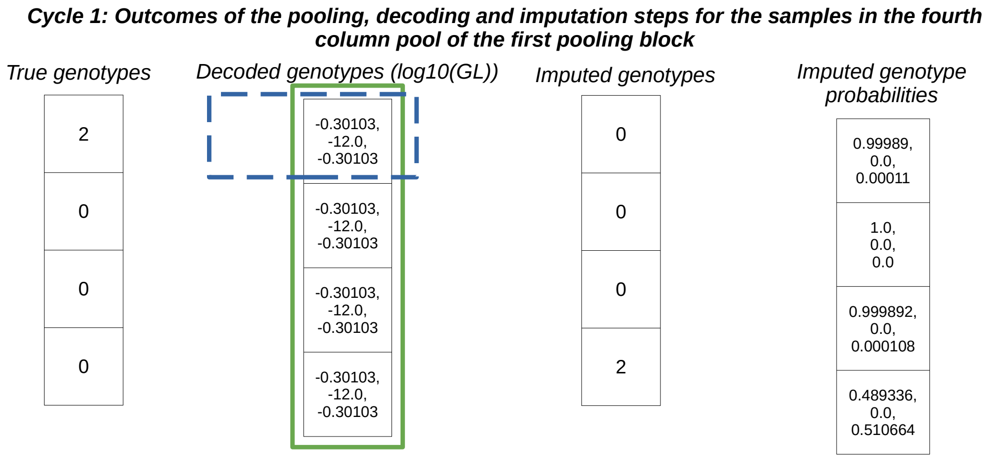

Figure S1.3: **True, pooled, decoded, and imputed genotype data for the pool P_8 in cycle 1.** We implemented a code that simulates error-free genotyping on the true data $(2, 0, 0, 0)$ and which returns the genotype 1 for the pool P_8. The imputed data after the cycle 1 is completed is $(0, 0, 0, 2)$, which matches the vector of true genotypes for all samples but G_4 and G_{16}. We note that the probability of the imputed genotype 2 for G_{16}, which is incorrect (concordance of 0.0), is close to 0.5, which means little confidence in the prediction and a low cross-entropy score. While the prediction for G_4 is incorrect as well (concordance of 0.0), the probability of the imputed genotype 0 is much higher (0.99989) and therefore the cross-entropy is larger as well.

Tables

	G_4		
True genotypes	0.0	0.0	1.0
Cycle 1			
Decoded genotypes	-0.30103	-12.0	-0.30103
Imputed genotypes	0.99989	0.0	0.00011
Cycle 2			
Decoded genotypes	-0.30103	-11.9933	-0.29431
Imputed genotypes	0.99989	0.0	0.00011
Cycle 11			
Decoded genotypes	-0.30103	-11.9135	-0.21449
Imputed genotypes	0.93886	0.0	0.06114
Cycle 12			
Decoded genotypes	-0.30103	-11.9074	-0.20846
Imputed genotypes	0.90776	0.0	0.09224
Cycle 22			
Decoded genotypes	-0.30103	-11.8706	-0.17167
Imputed genotypes	0.51456	0.0	0.48544
Cycle 23			
Decoded genotypes	-0.30103	-11.8868	-0.16922
Imputed genotypes	0.49122	0.0	0.50878
Cycle 41			
Decoded genotypes	-0.30102	-11.8337	-0.13472
Imputed genotypes	0.30713	0.0	0.69287
Cycle 42			
Decoded genotypes	-0.30101	-11.832	-0.13309
Imputed genotypes	0.30421	0.0	0.69579

Table S1.1: **Genotypes estimated from decoding and imputed for sample G_4 in the first pooling block at marker 1:11814388 through several cycles of pooling and imputation ($w = 0.01$).**
Decoded genotypes are given as genotype log10-likelihoods. True genotypes and imputed genotypes are given as genotypes probabilities. The underlined values in the decoded data signifies they were increased compared to the previous cycle, with a tolerance of 10^{-5} for the difference in likelihood. Red and green highlighting shades indicates whether the predicted genotype is correct or not. Darker shades are used for predictions with a probability close to 1.0 and lighter shades for weak predictions.
Sample G_4 in this example has the true genotype 1/1 (homozygote for the alternate allele), that is $(0.0, 0.0, 1.0)$ if written as genotype probabilities. Both alleles are detected in the pool P_1, such that after the first cycle of decoding, G_4 is equally likely to be one of the homozygotes. In the first round of imputation, G_4 is predicted as a homozygote for the reference allele with probability 0.99989, which is consistent with the observation for P_1 but incorrect with respect to the true data. As any sample in our overlapping design, G_4 is involved in another pool, which is P_8 in this case. For the pool P_8, `repool` identifies an inconsistency between the repooled imputed genotypes and the observations, thus the log10-likelihoods of the genotypes carrying the allele 1 in the previous decoded data are hence increased to $-12 + 0.0067 = -11.9933$ and $-0.30103 + 0.00672 = -0.29431$. These corrected data constitute the decoded data in cycle 2 and are passed as input to the imputation algorithm.
There is no noticeable improvement in the accuracy of the predicted genotype after cycle 2, but if keeping correcting the decoded genotype likelihoods through a few cycles, we observe a that the prediction for the wrong homozygote drops to 0.93886 after 11 cycles, and has decreased down to 0.30421 after 42 cycles. The imputed genotype swaps to a correct prediction in the cycle 23. Eventually, G_4 is imputed to the correct genotype with probability 0.69579.
In this particular example of sample and locus, we find evidence that iterating our strategy of combining genotype decoding from pooled data and imputation improves the genotyping accuracy.

Table S1.2: **Details of the calculations with `repool` in the pool P_1.**

Panel A: Calculation of the likelihood of each sequence of genotypes completing the pool P_1.

Sequence(s)	x_{P_1}	$z^{(1)}_{P_1}$	$\mathcal{L}(x_{P_1}; z^{(1)}_{P_1})$
(a)	(0, 0, 0, 0)<br>(2, 0, 0, 0)<br>(0, 2, 0, 2)	$\begin{pmatrix} 0.98753 \\ 0.00000 \\ 0.01247 \end{pmatrix}, \begin{pmatrix} 0.99974 \\ 0.00000 \\ 0.00026 \end{pmatrix}, \begin{pmatrix} 0.00000 \\ 0.00000 \\ 1.00000 \end{pmatrix}, \begin{pmatrix} 0.99989 \\ 0.00000 \\ 0.00011 \end{pmatrix}$	0.00000
(b)	(2, 0, 2, 0)		$0.01247 \times 0.99974 \times 1.00000 \times 0.99989 = 0.01247$
(c)	(2, 2, 2, 0)		$0.01247 \times 0.00026 \times 1.00000 \times 0.99989 \leq 0.00001$
(d)	(2, 0, 2, 2)		$0.01247 \times 0.99974 \times 1.00000 \times 0.00011 \leq 0.00001$
(e)	(0, 0, 2, 0)		$0.98753 \times 0.99974 \times 1.00000 \times 0.99989 = 0.98717$
(f)	(0, 2, 2, 0)		$0.98753 \times 0.00026 \times 1.00000 \times 0.99989 = 0.00025$
(g)	(0, 0, 2, 2)		$0.98753 \times 0.99974 \times 1.00000 \times 0.00011 = 0.00011$
(h)	(0, 2, 2, 2)		$0.98753 \times 0.00026 \times 1.00000 \times 0.00011 \leq 0.00001$
(i)	(2, 2, 2, 2)		$0.01247 \times 0.00026 \times 1.00000 \times 0.00011 \leq 0.00001$

Sequence (a): sequences with sample G_3 highlighted in grey show cases that have zero probability given the imputed results, they therefore do not affect the likelihood of detecting the allele 0. Note it is impossible not to detect A_1 because the imputed result for G_3 guarantees its detection. Similarly, any case where any of the sample is heterozygous are also inconsistent for the same reason as the GP imputed for genotype 1 is equal to 0.0. These cases are omitted for brevity: in theory, there are 3^4 distinct ordered sequences of genotypes, but there are only non-zero contributions for 8 of them (sequences (b) to (i)).

Sequence (i): The sequence of genotypes $(x_1, x_2, x_3, x_4) = (2, 2, 2, 2)$ is inconsistent as it has a pooled value equal to 2, whereas the actual genotype of the pool P_1 was assayed to 1. Given the imputed data, this sequence is extremely unlikely, so the resulting probability of detecting both alleles, consistent with the observation, is close to 1.

Panel B: Normalization of the likelihood of (not) detecting each allele in the pool P_1.

Sequence(s)	$\mathcal{L}(A_0)$	$\mathcal{L}(\bar{A_0})$	$\mathcal{L}(A_1)$	$\mathcal{L}(\bar{A_1})$
(a)	0.00000	0.00000	0.00000	0.00000
(b)	0.01247	0.00000	0.01247	0.00000
(c)	$\leq$ 0.00001	0.00000	$\leq$ 0.00001	0.00000
(d)	$\leq$ 0.00001	0.00000	$\leq$ 0.00001	0.00000
(e)	0.98717	0.00000	0.98717	0.00000
(f)	0.00025	0.00000	0.00025	0.00000
(g)	0.00011	0.00000	0.00011	0.00000
(h)	$\leq$ 0.00001	0.00000	$\leq$ 0.00001	0.00000
(i)	0.00000	$\leq$ 0.00001	$\leq$ 0.00001	0.00000

For the allele $A_0 = 0$: $Pr(A_0|z_{P_1}) = \frac{\sum_k \mathcal{L}(A_0)^{(k)}}{\sum_k \mathcal{L}(A_0)^{(k)} + \mathcal{L}(\bar{A_0})^{(k)}} = 0.99999$, which is inconsistent with the decoded data, therefore the decoded likelihoods for the genotypes $(A_0, A_0), (A_0, A_1)$ are corrected by $-log(0.99999) \cdot 0.01 \leq 0.00001$. This correction is too small for being significant and noticeable in the figures.

For the allele $A_1 = 1$: $Pr(A_1|z_{P_1}) = \frac{\sum_k \mathcal{L}(A_1)^{(k)}}{\sum_k \mathcal{L}(A_1)^{(k)} + \mathcal{L}(\bar{A_1})^{(k)}} = 1.0$, that is the decoded data is not updated for genotypes carrying allele A_1.

Table S1.3: **Details of the calculations with `repool` in the pool P_8.**

Panel A: Calculation of the likelihood of each sequence of genotypes completing the pool P_8.

Sequence(s)	x_{P_8}	$z^{(1)}_{P_8}$	$\mathcal{L}(x_{P_8}; z^{(1)}_{P_8})$
(a)	(0, 0, 0, 2)	$\begin{pmatrix}0.99989\\0.00000\\0.00011\end{pmatrix}, \begin{pmatrix}1.00000\\0.00000\\0.00000\end{pmatrix}, \begin{pmatrix}0.99989\\0.00000\\0.00011\end{pmatrix}, \begin{pmatrix}0.48934\\0.00000\\0.51066\end{pmatrix}$	0.510553
(b)	(0, 0, 2, 2)		0.00006
(c)	(2, 0, 2, 2)		$\leq$ 0.00001
(d)	(0, 0, 2, 0)		0.00005
(e)	(2, 0, 0, 0)		0.00005
(f)	(2, 0, 2, 0)		$\leq$ 0.00001
(g)	(2, 0, 0, 2)		0.00006
(h)	(0, 0, 0, 0)		0.48229

We proceed for P_8 similarly as in Table S1.2 with P_1.

Panel B: Normalization of the likelihood of (not) detecting each allele in the pool P_8.

Sequence(s)	$\mathcal{L}(A_0)$	$\mathcal{L}(\bar{A}_0)$	$\mathcal{L}(A_1)$	$\mathcal{L}(\bar{A}_1)$
(a)	0.51055	0.00000	0.51055	0.00000
(b)	0.00006	0.00000	0.00006	0.00000
(c)	$\leq$ 0.00001	0.00000	$\leq$ 0.00001	0.00000
(d)	0.00005	0.00000	0.00005	0.00000
(e)	0.00005	0.00000	0.00005	0.00000
(f)	$\leq$ 0.00001	0.00000	$\leq$ 0.00001	0.00000
(g)	0.00006	0.00000	0.00006	0.00000
(h)	0.48229	0.00000	0.00000	0.48229

For the allele $A_0 = 0$: $Pr(A_0|z_{P_8}) = 1.0$, hence no correction is applied.
For the allele $A_1 = 1$: $Pr(A_1|z_{P_1}) = 0.51077$, that is a correction of $-log(0.51077) \cdot 0.01 = +0.00672$ is applied for the genotypes carrying allele A_1. This is the actual increase in likelihood we observe in the decoded data between cycle 1 and cycle 2 for sample G_4 in Table S1.1.
