# Supplementary material for: Using feedback in pooled experiments augmented with imputation for high genotyping accuracy at reduced cost
Source: G3 (Bethesda). 2025 Jan 23;15(3):jkaf010. doi: 10.1093/g3journal/jkaf010 (PMC11917477; doi:10.1093/g3journal/jkaf010)
Supplement: jkaf010_Supplementary_Data [file jkaf010_supplementary_data.zip › File_S2_G3-2024-405589.pdf]

Combining pooling and imputation in a feedback structure for SNP genotyping at reduced cost and increased accuracy

Camille Clouard [1], Carl Nettelblad [1,2*]

[1] Division of Scientific Computing, Department of Information Technology, Uppsala University, Lägerhyddsvägen 1, 75237, Uppsala, Sweden
[2] SciLifeLab, Science for Life Laboratory, Husargatan 3, 75237, Uppsala, Sweden
Corresponding author: carl.nettelblad@it.uu.se

Supplemental file 2:

- Number of pages: 11
- Number of figures: 10
- Number of tables: 1

Supplemental figures

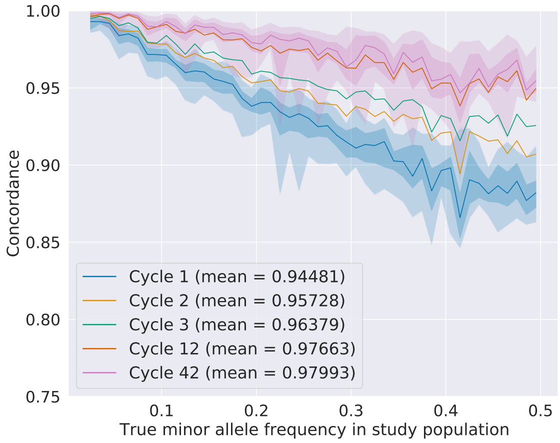

(a) **Concordance after cycles 1, 2, 3, 12, and 42.**

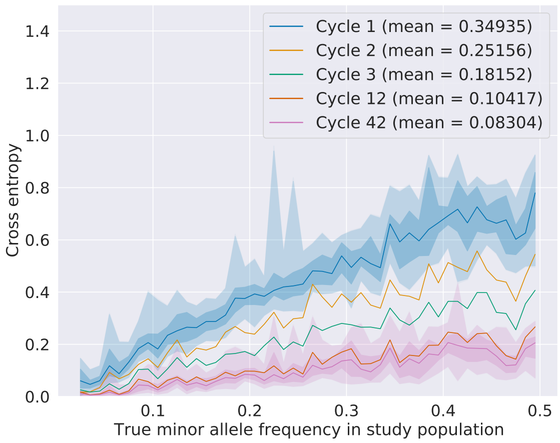

(b) **Cross-entropy after cycles 1, 2, 3, 12, and 42.**

Figure S2.1: **Genotyping accuracy after cycles 1, 2, 3, 12, and 42 (correction factor $w = 0.05$).**

The concordance and cross-entropy scores are computed between the imputed data and the filtered data. The imputed markers are sorted per ascending true MAF in the study population and categorized into MAF bins (bin size equal to 0.01). Each marker has a smoothed concordance and cross-entropy score which is calculated as the average value in a rolling window of 5 MAF-consecutive markers. The plain line shows the median accuracy value (concordance or cross-entropy) in each MAF bin, and the shadowed areas represent the quantiles $[0.0, 0.01, 0.25, 0.75, 0.99, 1.0]$. For the sake of readability, the envelopes for the quantiles are shown only for the first and the last cycles. The irregularities of the median line and of the envelopes, for instance around $MAF \sim 0.22$, are likely due to the sparsity of the markers in combination to the small width of the window used for rolling averaging.

We observe the strongest improvement in genotyping accuracy for variants with $MAF \geq 0.3$ and through the first cycles (2 and 3).

S2.1a: Concordance computed for all genotypes.

S2.1b: Cross-entropy computed for all genotypes.

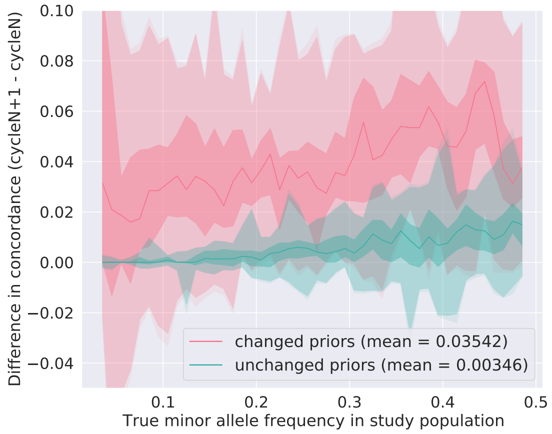

(a) **Improvement in concordance (N=1).**

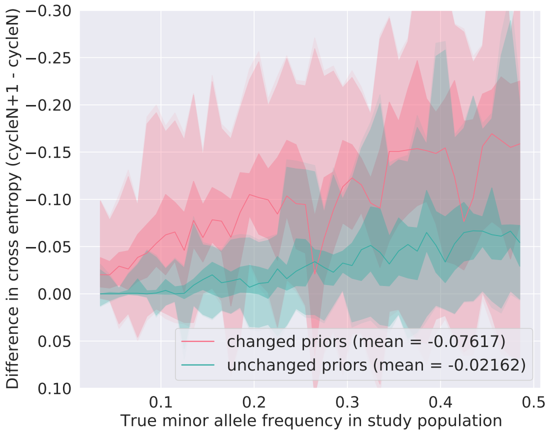

(b) **Improvement in cross-entropy (N=1).**

Figure S2.2: **Improvement in genotyping accuracy between cycle 1 and cycle 2 (N=1) computed separately for the genotypes with an *updated* prior and for the genotypes with an *unchanged* prior (correction factor $w = 0.05$).**
The plain line shows the median accuracy score in each MAF bin. The shadowed areas display the quantiles $[0.0, 0.01, 0.25, 0.75, 0.99, 1.0]$. The per-marker averaged concordance and the cross-entropy scores for the genotypes with (un)changed priors are calculated in rolling windows of width 20.
Overall, the largest gain in accuracy is obtained for the set of genotypes whose priors were corrected, but an additional round of imputation also improves, to a smaller extent, the genotyping accuracy for the predicted genotypes that were consistent with the pooled outcomes and that were therefore not changed.
S2.2a: Positive values indicate that the concordance achieved after the cycle N+1 is higher than the concordance after cycle N, that is, the iteration N+1 has improved the genotyping accuracy.
S2.2b: Negative values indicate that the cross-entropy achieved after the cycle N+1 is lower than the concordance after cycle N, that is, the iteration N+1 has improved the genotyping accuracy. The y-axis is reverted in order to display the improvements above the origin.

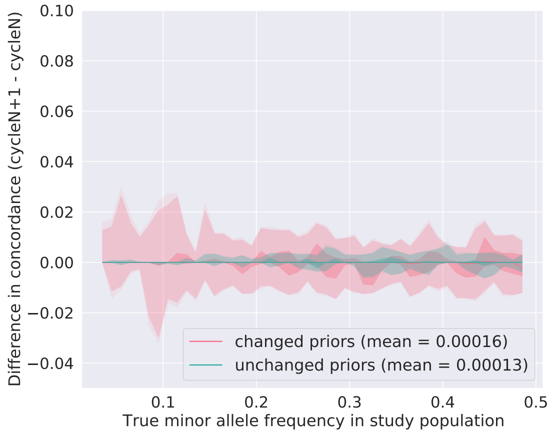

(a) **Improvement in concordance (N=11).**

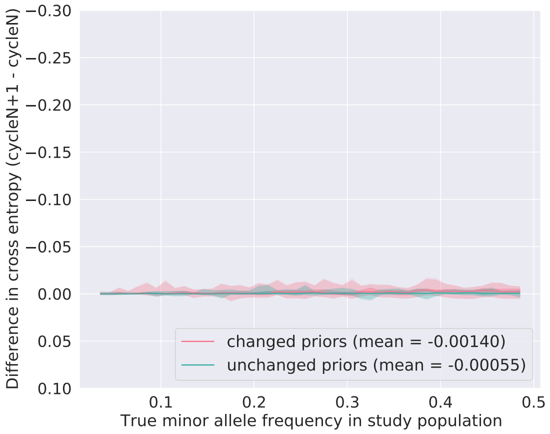

(b) **Improvement in cross-entropy (N=11).**

Figure S2.3: **Improvement in genotyping accuracy between cycle 12 and cycle 11 (N=11) computed separately for the genotypes with an *updated* prior and for the genotypes with an *unchanged* prior (correction factor $w = 0.05$).**
The plain line shows the median accuracy score in each bin. The shadowed areas display the quantiles $[0.0, 0.01, 0.25, 0.75, 0.99, 1.0]$.
The gain in accuracy between consecutive cycles has strongly decreased.
S2.3a: For the set of changed entries, the peaks observed around $MAF = 0.1$ and $MAF = 0.04$ suggest an instability in some weak genotype predictions.
S2.3b: The tables below show an example of a true genotype value that is changed to the incorrect homozygote in cycle 12 (detrimental swap), and an example of a true genotype value that is changed correctly (favorable swap). We do observe homozygous genotype probabilities that are close to 0.5.

Example of "detrimental last swap in genotype prediction with nearly equally likely GP for the opposite homozygotes:
Sample A1447_A1447 at variant 1:269993167 ($MAF = 0.098790$) has GT = 0/0,
which is correctly predicted after cycle 11 but is predicted to 1/1 in cycle 12.

	Cycle 11	Cycle 12
GT:GP	0/0:0.536581,0,0.463419	1/1:0.499091,0,0.500909
Concordance	1.0	0.0
Difference cycle 12 - cycle 11	+1.0	
Cross-entropy	5.33531	5.76693
Difference cycle 12 - cycle 11	-0.43162	

Example of favorable last swap in genotype prediction with nearly equally likely GP for the opposite homozygotes:
Sample A1226_A1226 at variant 1:4054091 ($MAF = 0.034274$) has GT = 1/1,
which is incorrectly predicted to 0/0 after cycle 11 but rectified to 1/1 in cycle 12.

	Cycle 11	Cycle 12
GT:GP	0/0:0.514225,0,0.485775	1/1:0.466806,0,0.533194
Concordance	0.0	1.0
Difference cycle 12 - cycle 11	-1.0	
Cross-entropy	5.92023	5.37430
Difference cycle 12 - cycle 11	+0.54593	

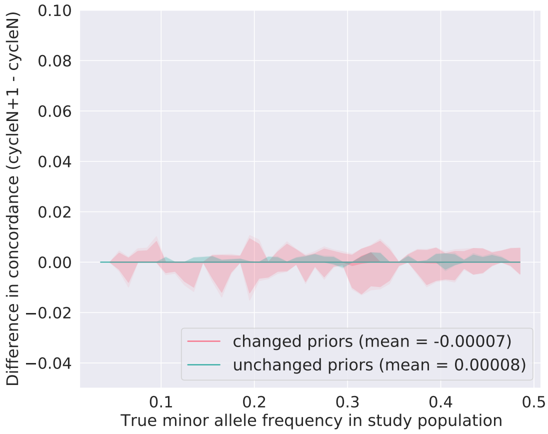

(a) **Improvement in concordance (N=41).**

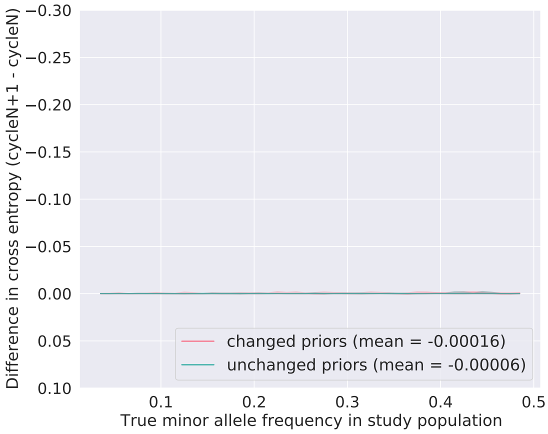

(b) **Improvement in cross-entropy (N=41).**

Figure S2.4: **Improvement in genotyping accuracy between cycle 42 and cycle 41 (N=41) computed separately for the genotypes with an *updated* prior and for the genotypes with an *unchanged* prior (correction factor $w = 0.05$).**
The plain line shows the median accuracy score in each bin. The shadowed areas display the quantiles $[0.0, 0.01, 0.25, 0.75, 0.99, 1.0]$.
We no longer observe any significant gain in accuracy, that is there is a convergence phenomenon in both sets of genotypes with (un)corrected priors.
S2.4a: The average Improvement in concordance is slightly positive for the markers whose prior was corrected, which means that the cycle 42 worsen the results compared with cycle 41.
S2.4b: Cross-entropy is barely modified, which could indicate that the genotypes that were incorrectly swapped compared to the cycle 41 have GP that are close to equiprobability for the two homozygotes, as the examples shown in Figure S2.3.

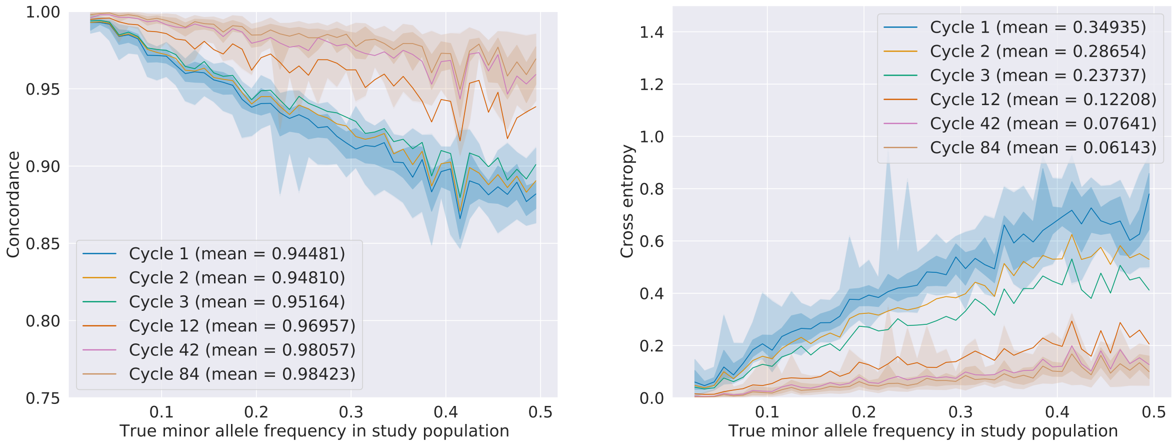

(a) **Concordance.**

(b) **Cross-entropy.**

Figure S2.5: **Genotyping accuracy after cycles 1, 2, 3, 12, 42, and 84 (correction factor $w = 0.005$).**

The concordance and cross-entropy scores are computed between the imputed data and the filtered data. The imputed markers are sorted per ascending true MAF in the study population and categorized into MAF bins (bin size equal to 0.01). Each marker has a smoothed concordance and cross-entropy score which is calculated as the average value in a rolling window of 5 MAF-consecutive markers. The plain line shows the median accuracy value (concordance or cross-entropy) in each MAF bin, and the shadowed areas represent the quantiles $[0.0, 0.01, 0.25, 0.75, 0.99, 1.0]$. For the sake of readability, the envelopes for the quantiles are shown only for the first and the last cycles. The irregularities of the median line and of the envelopes, for instance around $MAF \sim 0.22$, are likely due to the sparsity of the markers in combination to the small width of the window used for rolling averaging.

We observe the strongest improvement in genotyping accuracy for variants with $MAF \geq 0.3$ and through the first cycles (2 and 3).

S2.5a: Concordance computed for all genotypes.

S2.5b: Cross-entropy computed for all genotypes.

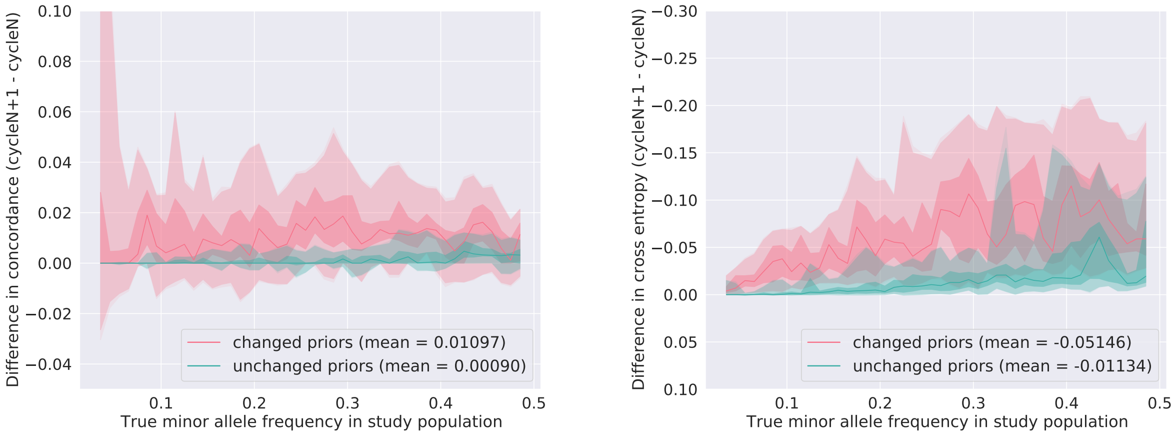

(a) **Improvement in concordance (N=1).**

(b) **Improvement in cross-entropy (N=1).**

Figure S2.6: **Improvement in genotyping accuracy between cycle 1 and cycle 2 (N=1) computed separately for the genotypes with an *updated* prior and for the genotypes with an *unchanged* prior (correction factor $w = 0.005$).**

The plain line shows the median accuracy score in each MAF bin. The shadowed areas display the quantiles $[0.0, 0.01, 0.25, 0.75, 0.99, 1.0]$. The per-marker averaged concordance and the cross-entropy scores for the genotypes with (un)changed priors are calculated in rolling windows of width 20.

Overall, the largest gain in accuracy is obtained for the set of genotypes whose priors were corrected, but an additional round of imputation also improves, to a smaller extent, the genotyping accuracy for the predicted genotypes that were consistent with the pooled outcomes and that were therefore not changed.

S2.6a: Positive values indicate that the concordance achieved after the cycle N+1 is higher than the concordance after cycle N, that is, the iteration N+1 has improved the genotyping accuracy.

S2.6b: Negative values indicate that the cross-entropy achieved after the cycle N+1 is lower than the cross-entropy after cycle N, that is, the iteration N+1 has improved the genotyping accuracy. The y-axis is reverted for facilitating the interpretation.

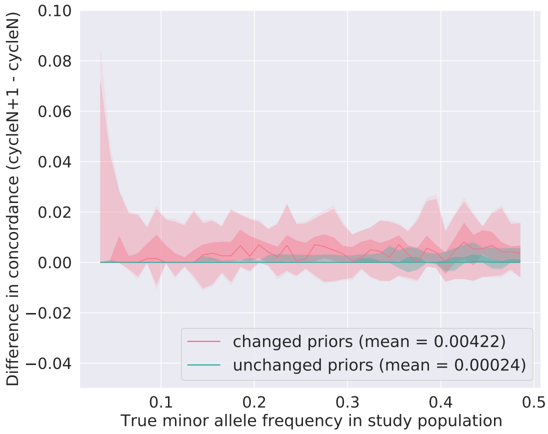

(a) **Improvement in concordance (N=11).**

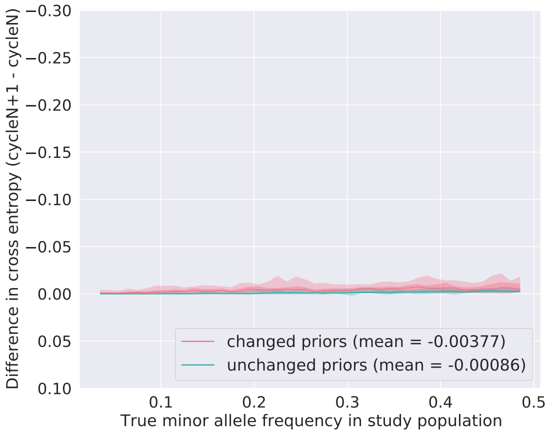

(b) **Improvement in cross-entropy (N=11).**

Figure S2.7: **Improvement in genotyping accuracy between cycle 11 and cycle 12 (N=11) computed separately for the genotypes with an *updated* prior and for the genotypes with an *unchanged* prior (correction factor $w = 0.005$).**
The plain line shows the median accuracy score in each bin. The shadowed areas display the quantiles $[0.0, 0.01, 0.25, 0.75, 0.99, 1.0]$.
S2.7a: Positive values indicate that the concordance achieved after the cycle N+1 is higher than the concordance after cycle N, that is, the iteration N+1 has improved the genotyping accuracy.
S2.7b: Negative values indicate that the cross-entropy achieved after the cycle N+1 is lower than the concordance after cycle N, that is, the iteration N+1 has improved the genotyping accuracy.

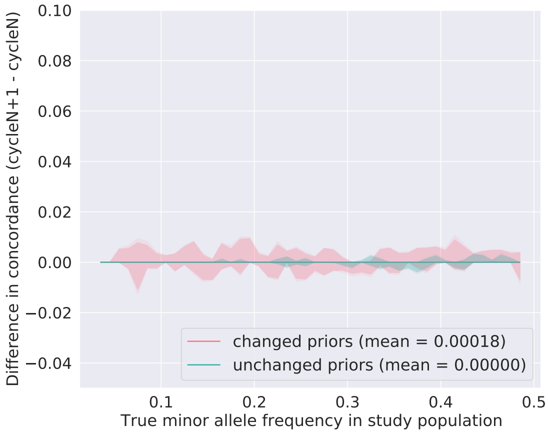

(a) **Improvement in concordance (N=41).**

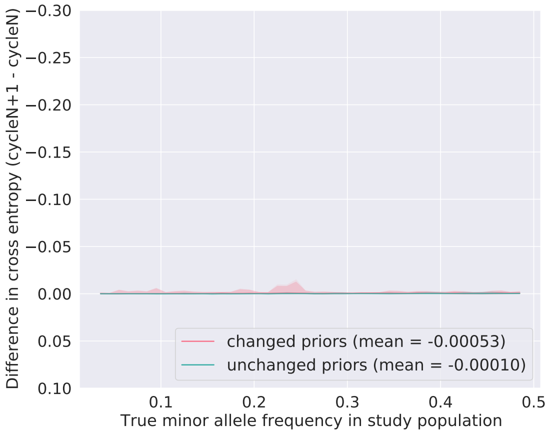

(b) **Improvement in cross-entropy (N=41).**

Figure S2.8: **Improvement in genotyping accuracy between cycle 41 and cycle 42 (N=41) computed separately for the genotypes with an *updated* prior and for the genotypes with an *unchanged* prior (correction factor $w = 0.005$).**
The plain line shows the median accuracy score in each bin. The shadowed areas display the quantiles $[0.0, 0.01, 0.25, 0.75, 0.99, 1.0]$.
S2.8a: The overall average Improvement in concordance for the set of changed entries is slightly negative which indicates that some additional cycles could increase the accuracy, but the positive pink upper-envelope also shows some loss in concordance.
S2.8b: Cross-entropy is barely modified, which could indicate that the improvements achieved at some variants are counterbalanced by other predictions that are worsened.

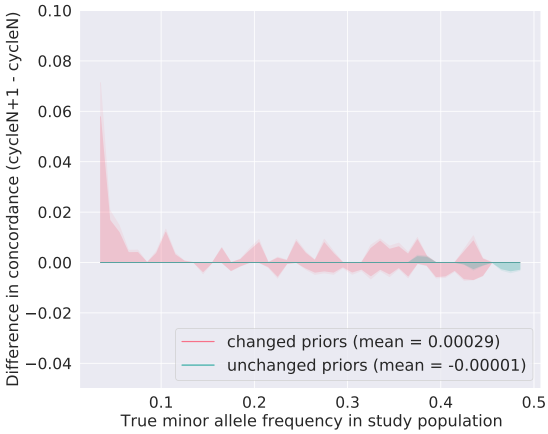

(a) **Improvement in concordance (N=83).**

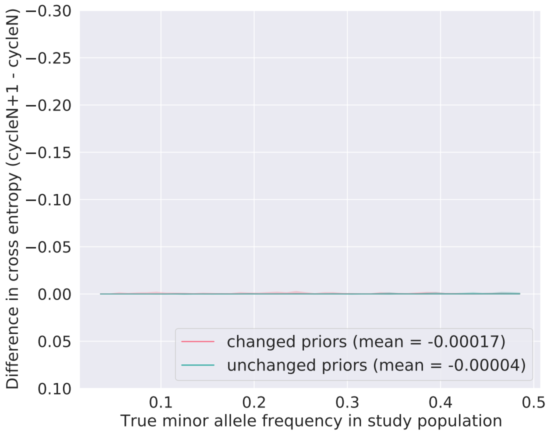

(b) **Improvement in cross-entropy (N=83).**

Figure S2.9: **Improvement in genotyping accuracy between cycle 83 and cycle 84 (N=83) computed separately for the genotypes with an *updated* prior and for the genotypes with an *unchanged* prior (correction factor $w = 0.005$).**
The plain line shows the median accuracy score in each bin. The shadowed areas display the quantiles $[0.0, 0.01, 0.25, 0.75, 0.99, 1.0]$.
S2.9a: The average Improvement in concordance is slightly positive for the markers whose prior was not corrected, which means that the cycle 84 worsen the results compared with cycle 41. Genotyping accuracy no longer increases in spite of running additional cycles.
S2.9b: Cross-entropy is barely modified, convergence is reached.

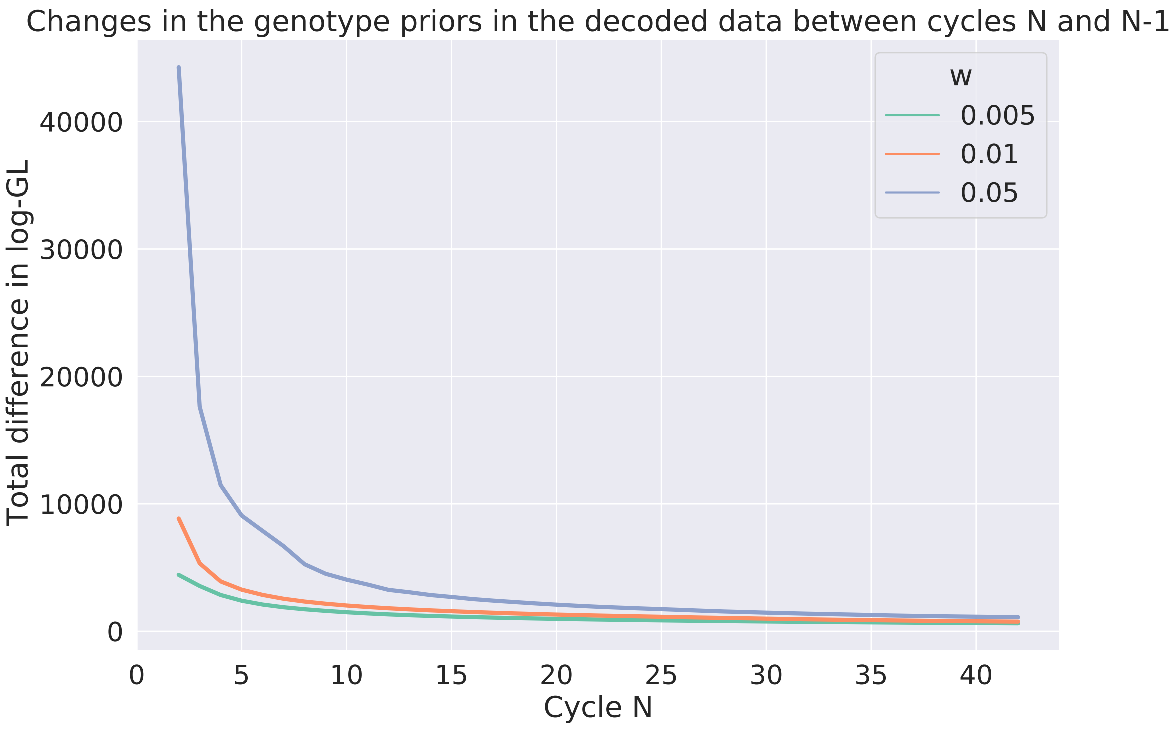

Figure S2.10: **Trajectories of the total correction made in the decoded data for various correction factors w.**
The total log-GL difference renders the information gained from imputing the data, that is how much the genetic structure at the population level can enrich the outcomes from pooling.
Number of values used for computing the total difference: 496 samples x 1170 markers = 580,320 genotypes.
The difference in log-GL between the pooled-decoded data in the cycles N and N-1 for the sample i sample is computed as $\sum_{g=0}^{g=2} y(g)_i^{(N)} - y(g)_i^{(N-1)}$, with the same notations as in Algorithm 1 in the main manuscript and g as an integer-valued genotype. The largest values of the total difference in log-GL are observed between the cycles 1 and 2 ($N = 2$) with 44267 ($w = 0.05$), 8853 ($w = 0.01$), and 4427 ($w = 0.005$). That is, the total correction is directly proportional to w in the second iteration. The smallest values of the total difference in log-GL are observed between the second last and the last cycle with 1103 ($w = 0.05$, from cycle 41 to cycle 42), 756 ($w = 0.01$, from cycle 41 to cycle 42), and 624 ($w = 0.005$, from cycle 41 to cycle 42).
Note: $w = 0.005$ is half of $w = 0.01$ and for investigating if our method has some linear behavior, we therefore execute twice as many cycles with $w = 0.005$ (84 cycles) as with $w = 0.01$ (42 cycles). For readability, only 42 cycles are displayed in both series.
As expected, the total correction of the genotype likelihoods made between consecutive cycles increases with the correction factor, and the trajectory shows stronger convergence for larger values of w. In other words, smaller values for the correction factor lead to more cycles to be run before convergence is observed.

Supplemental tables

Table S2.1: Statistics for genotypes (variants x samples) per MAF bin in the population of inbred lines (pooled data, correction factor for the pooled genotype likelihoods equal to 0.05)

	0.00-0.05	0.05-0.10	0.10-0.20	0.20-0.30	0.30-0.40	0.40-0.50	Total
All cycles: pooled data							
Counts	37696	128960	180048	109616	68944	55056	580320
Proportions[1]	0.065	0.222	0.310	0.189	0.119	0.095	1.000
Cycle 2 vs. cycle 1:							
genotypes with updated priors in pooled data							
Counts	2316	18480	49662	39360	27268	22500	159586
Proportions w.r.t. the bin	0.061	0.143	0.276	0.359	0.396	0.409	0.275
Cycle 3 vs. cycle 2:							
genotypes with updated priors in pooled data							
Counts	2147	14916	47902	40302	30812	27502	163581
Proportions w.r.t. the bin	0.057	0.116	0.266	0.368	0.447	0.500	0.282
Cycle 12 vs. cycle 11:							
genotypes with updated priors in pooled data							
Counts	1968	13983	42939	36175	27456	24294	146815
Proportions w.r.t. the bin	0.052	0.108	0.238	0.330	0.398	0.441	0.253

[1] SNPs proportions per MAF bin with respect to the total number of SNPs on the genetic map.
